# Crystal structure of calcium dinickel(II) iron(III) tris­(orthophosphate): CaNi_2_Fe(PO_4_)_3_

**DOI:** 10.1107/S2056989017007411

**Published:** 2017-05-26

**Authors:** Said Ouaatta, Abderrazzak Assani, Mohamed Saadi, Lahcen El Ammari

**Affiliations:** a Laboratoire de Chimie du Solide Appliquée, Faculty of Sciences, Mohammed V University in Rabat, Avenue Ibn Battouta, BP 1014, Rabat, Morocco

**Keywords:** crystal structure, CaNi_2_Fe(PO_4_)_3_, transition metal phosphate, solid-state reactions, α-CrPO_4_ structure type

## Abstract

The title compound, CaNi_2_Fe(PO_4_)_3_, was synthesized by solid-state reactions. Its structure is closely related to that of α-CrPO_4_ in the space group *Imma*. Except for two O atoms in general positions, all atoms are located in special positions. The three-dimensional framework is built up from two types of sheets extending parallel to (100). The first sheet is made up from two edge-sharing [NiO_6_] octa­hedra, leading to the formation of [Ni_2_O_10_] double octa­hedra that are connected to two PO_4_ tetra­hedra through a common edge and corners. The second sheet results from rows of corner-sharing [FeO_6_] octa­hedra and PO_4_ tetra­hedra forming an infinite linear chain. These layers are linked together through common corners of PO_4_ tetra­hedra and [FeO_6_] octa­hedra, resulting in an open three-dimensional framework that delimits two types of channels parallel to [100] and [010] in which the eightfold-coordinated Ca^II^ cations are located.

## Abstract 2

The transition metal orthophosphate CaNi_2_Fe(PO_4_)_3_ adopts the α-CrPO_4_ structure type. The structure is built up from two types of sheets, resulting in an open three-dimensional framework that delimits two types of channels in which the Ca^II^ cations are located.

## Chemical context   

Phosphates belonging to the alluaudite (Moore, 1971 ▸) or to the *α*-CrPO_4_ (Attfield *et al.*, 1988 ▸) structure type exhibit inter­esting physical and chemical properties. Consequently, these compounds have many promising applications such as use as positive electrodes in lithium and sodium batteries (Kim *et al.*, 2014 ▸; Huang *et al.*, 2015 ▸) or as catalysts (Kacimi *et al.*, 2005 ▸). Over the last few years, phosphate-based compounds crystallizing in the α-CrPO_4_ or alluaudite structure types have been investigated by us. In this context, new phosphates adopting the alluaudite or α-CrPO_4_ structure type have been synthesized and structurally characterized. For example, the mixed-valence manganese phosphates PbMn^II^_2_Mn^III^(PO_4_)_3_ (Alhakmi *et al.*, 2013 ▸) and PbMn^II^_2_Mn^III^(PO_4_)_3_ (Assani *et al.*, 2013 ▸), the magnesium phosphate NaMg_3_(PO_4_)(HPO_4_)_2_ (Ould Saleck *et al.*, 2015 ▸) and silver nickel phosphate Ag_2_Ni_3_(HPO_4_)(PO_4_)_2_ (Assani *et al.*, 2011 ▸) were synthesized by hydro­thermal methods, while solid-state reactions were applied to synthesize SrNi_2_Fe(PO_4_)_3_ (Ouaatta *et al.*, 2015 ▸) and Na_2_Co_2_Fe(PO_4_)_3_ (Bouraima *et al.*, 2015 ▸). In a continuation of the latter preparation route, we have investigated pseudo-quaternary systems *M*O–NiO–Fe_2_O_3_–P_2_O_5_ (*M* represents a divalent cation) and report here on the synthesis and crystal structure of the title compound, CaNi_2_Fe(PO_4_)_3_.

## Structural commentary   

**Figure 1:**
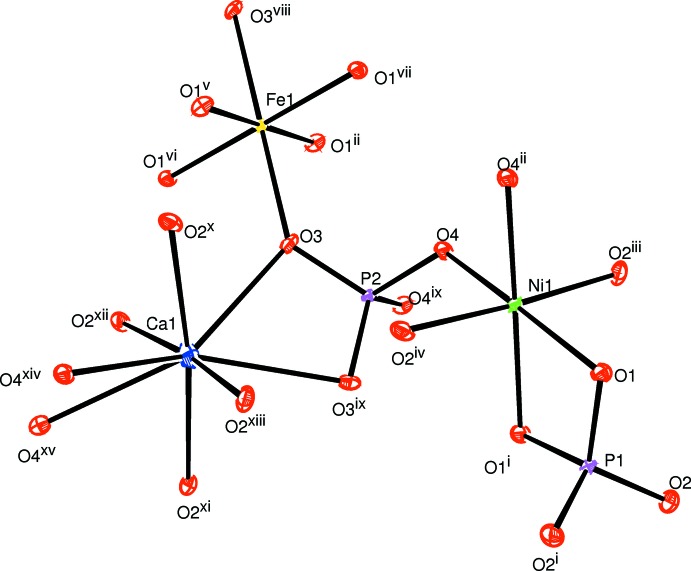
The principal building units in the crystal structure of the title compound. Displacement ellipsoids are drawn at the 50% probability level. [Symmetry codes: (i) −*x* + 2, −*y* + 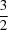, *z* + 1; (ii) *x*, *y*, *z* + 1; (iii) −*x* + 2, −*y* + 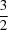, *z*; (iv) −*x* + 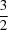, −*y* + 1, *z* + 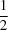; (v) *x* + 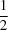, *y* + 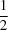, *z* + 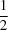; (vi) −*x* + 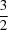, *y* + 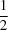, *z* + 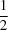; (vii) *x* + 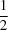, −*y* + 1, *z* + 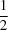; (viii) −*x* + 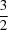, −*y* + 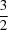, −*z* + 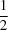; (ix) −*x* + 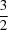, *y*, −*z* + 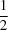; (*x*) *x*, −*y* + 1, −*z*; (xi) −*x* + 1, *y*, *z*; (xii) *x*, −*y* + 1, −*z* + 1; (xiii) −*x* + 1, −*y* + 1, −*z* + 1; (xiv) *x* − 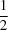, *y*, −*z* + 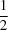.]

**Figure 2:**
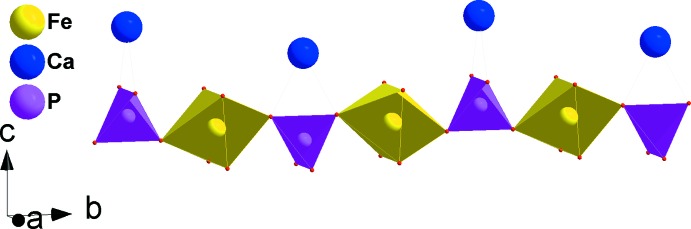
A chain formed by sharing corners of PO_4_ tetra­hedra and [FeO_6_] octa­hedra, alternating with a zigzag chain of calcium cations.

**Figure 3:**
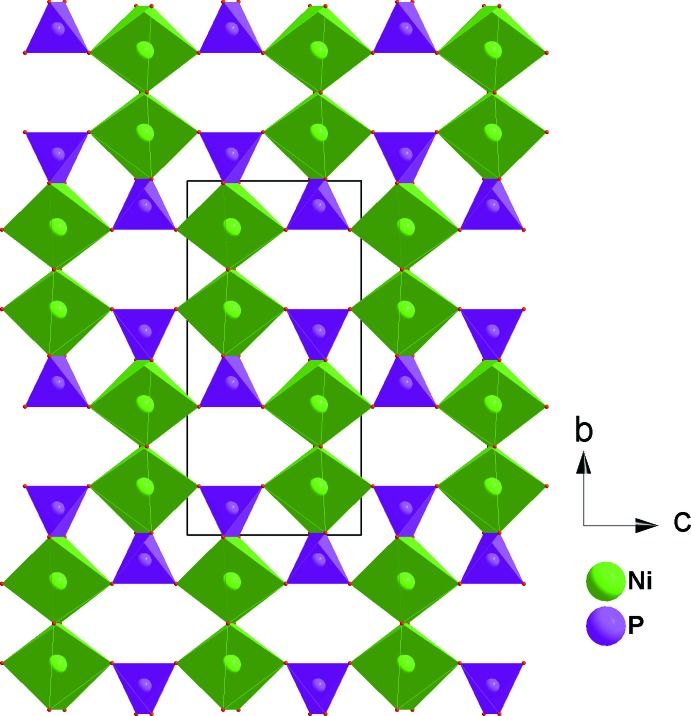
Edge-sharing [NiO_6_] octa­hedra linked by PO_4_ tetra­hedra, forming a sheet parallel to (100).

**Figure 4:**
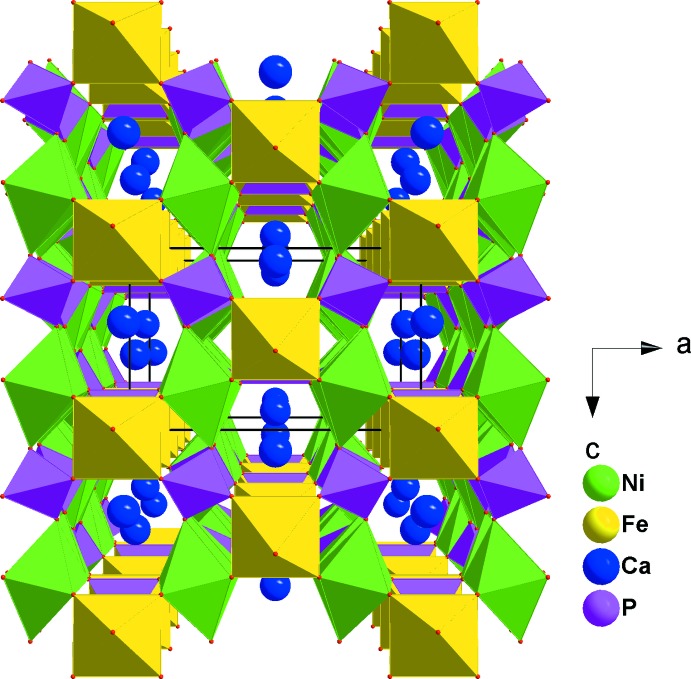
Polyhedral representation of CaNiO_2_Fe(PO_4_)_3_, showing channels running parallel to [100].

CaNi_2_Fe(PO_4_)_3_ crystallizes in the α-CrPO_4_ structure type. The principal building units of the crystal structure are one [CaO_8_] polyhedron, [FeO_6_] and [NiO_6_] octa­hedra and PO_4_ tetra­hedra, as shown in Fig. 1 ▸.The octa­hedral coordination sphere of the iron(III) cation is more distorted than that of nickel(II), with Fe—O bond lengths in the range 1.9504 (7)–2.0822 (11) Å and Ni—O bond lengths in the range 2.0498 (8)–2.0841 (8) Å. In the title structure, all atoms are on special positions, except for the two oxygen atoms O1 and O2, which are on general positions. The structure can be described by the stacking of two types of sheets extending parallel to (100). The first sheet is formed by alternating [FeO_6_] octa­hedra and PO_4_ tetra­hedra sharing corners to build a linear infinite chain surrounding a zigzag chain of Ca^II+^ cations (Fig. 2 ▸). The second sheet is built up from two edge-sharing [NiO_6_] octa­hedra leading to the formation of [Ni_2_O_10_] double octa­hedra, which are connected to two PO_4_ tetra­hedra by a common edge and a common corner, as shown in Fig. 3 ▸. The linkage of both layers, through vertices of PO_4_ tetra­hedra and [FeO_6_] octa­hedra, gives rise to the formation of an open three-dimensional framework that delimits two types of channels parallel to [100] and [010] in which the Ca^II^ cations are located with eight neighbouring O atoms, as shown in Fig. 4 ▸. The title compound has a stoichiometric composition like that of the related strontium homologue SrNi_2_Fe(PO_4_)_3_.

## Synthesis and crystallization   

CaNi_2_Fe(PO_4_)_3_ was prepared by solid-state reactions in air. Stoichiometric mixtures of calcium, nickel and iron precursors were dissolved in water to which 85%_wt_ phospho­ric acid was added. The obtained mixture was stirred without heating for 24 h and was subsequently evaporated to dryness at 343 K. The resulting dry residue was ground in an agate mortar until homogeneity, progressively heated in a platinum crucible up to 873 K to remove the volatile decomposition products, and then melted at 1433 K. The molten product was cooled down slowly with a 5 K h^−1^ rate and then to room temperature. The crystals obtained after washing with water were orange with parallelepipedal forms.

## Refinement   

Crystal data, data collection and structure refinement details are summarized in Table 1 ▸. The maximum and minimum remaining electron densities are 0.68 and 0.41 Å, respectively, away from the Ni1 site.

## Supplementary Material

Crystal structure: contains datablock(s) I. DOI: 10.1107/S2056989017007411/wm5390sup1.cif

Structure factors: contains datablock(s) I. DOI: 10.1107/S2056989017007411/wm5390Isup2.hkl

CCDC reference: 1551182

Additional supporting information:  crystallographic information; 3D view; checkCIF report

## Figures and Tables

**Table 1:** Experimental details

Crystal data	
Chemical formula	CaNi_2_Fe(PO_4_)_3_
*M*_r_	498.26
Crystal system, space group	Orthorhombic, *I* *m* *m* *a*
Temperature (K)	296
*a*, *b*, *c* (Å)	10.3126 (3), 13.1138 (3), 6.4405 (2)
*V* (Å^3^)	871.00 (4)
*Z*	4
Radiation type	Mo *K*α
μ (mm^−1^)	7.14
Crystal size (mm)	0.30 × 0.27 × 0.21

Data collection	
Diffractometer	Bruker X8 APEX
Absorption correction	Multi-scan (*SADABS*; Krause *et al.*, 2015 ▸)
*T*_min_, *T*_max_	0.596, 0.748
No. of measured, independent and observed [*I* > 2σ(*I*)] reflections	8446, 1171, 1153
*R*_int_	0.020
(sin θ/λ)_max_ (Å^−1^)	0.840

Refinement	
*R*[*F* ^2^ > 2σ(*F* ^2^)], *wR*(*F* ^2^), *S*	0.017, 0.044, 1.17
No. of reflections	1171
No. of parameters	54
Δρ_max_, Δρ_min_ (e Å^−3^)	0.76, −0.63

Computer programs: *APEX2* and *SAINT* (Bruker, 2009 ▸), *SHELXT2014* (Sheldrick, 2015*a* ▸), *SHELXL2014* (Sheldrick, 2015*b* ▸), *ORTEP-3 for Windows* (Farrugia, 2012 ▸), *DIAMOND* (Brandenburg, 2006 ▸) and *publCIF* (Westrip, 2010 ▸).

## supplementary crystallographic information

### Crystal data

**Table:**

CaNi_2_Fe(PO_4_)_3_	*D*_x_ = 3.800 Mg m^−^^3^
*M**_r_* = 498.26	Mo *K*α radiation, λ = 0.71073 Å
Orthorhombic, *I**m**m**a*	Cell parameters from 1171 reflections
*a* = 10.3126 (3) Å	θ = 3.1–36.6°
*b* = 13.1138 (3) Å	µ = 7.14 mm^−^^1^
*c* = 6.4405 (2) Å	*T* = 296 K
*V* = 871.00 (4) Å^3^	Parallelepiped, orange
*Z* = 4	0.30 × 0.27 × 0.21 mm
*F*(000) = 972	

### Data collection

**Table:**

Bruker X8 APEX diffractometer	1171 independent reflections
Radiation source: fine-focus sealed tube	1153 reflections with *I* > 2σ(*I*)
Graphite monochromator	*R*_int_ = 0.020
φ and ω scans	θ_max_ = 36.6°, θ_min_ = 3.1°
Absorption correction: multi-scan (SADABS; Krause *et al.*, 2015)	*h* = −16→17
*T*_min_ = 0.596, *T*_max_ = 0.748	*k* = −20→22
8446 measured reflections	*l* = −10→10

### Refinement

**Table:**

Refinement on *F*^2^	0 restraints
Least-squares matrix: full	*w* = 1/[σ^2^(*F*_o_^2^) + (0.0216*P*)^2^ + 1.467*P*] where *P* = (*F*_o_^2^ + 2*F*_c_^2^)/3
*R*[*F*^2^ > 2σ(*F*^2^)] = 0.017	(Δ/σ)_max_ = 0.001
*wR*(*F*^2^) = 0.044	Δρ_max_ = 0.76 e Å^−^^3^
*S* = 1.17	Δρ_min_ = −0.63 e Å^−^^3^
1171 reflections	Extinction correction: SHELXL2014 (Sheldrick, 2015*b*), Fc^*^=kFc[1+0.001xFc^2^λ^3^/sin(2θ)]^-1/4^
54 parameters	Extinction coefficient: 0.0033 (2)

### Special details

**Table:**

Geometry. All esds (except the esd in the dihedral angle between two l.s. planes) are estimated using the full covariance matrix. The cell esds are taken into account individually in the estimation of esds in distances, angles and torsion angles; correlations between esds in cell parameters are only used when they are defined by crystal symmetry. An approximate (isotropic) treatment of cell esds is used for estimating esds involving l.s. planes.

### Fractional atomic coordinates and isotropic or equivalent isotropic displacement parameters (Å^2^)

**Table:**

	*x*	*y*	*z*	*U*_iso_*/*U*_eq_	
Ni1	0.7500	0.36655 (2)	0.7500	0.00475 (5)	
Fe1	0.5000	0.0000	0.5000	0.00372 (6)	
Ca1	0.5000	0.2500	0.08981 (7)	0.01187 (8)	
P1	0.7500	0.57298 (3)	0.7500	0.00385 (7)	
P2	0.5000	0.2500	0.58291 (8)	0.00327 (8)	
O1	0.86146 (7)	0.49415 (6)	0.79418 (13)	0.00590 (12)	
O4	0.61754 (11)	0.2500	0.73284 (17)	0.00587 (16)	
O3	0.5000	0.15625 (8)	0.44256 (18)	0.00672 (17)	
O2	0.70724 (8)	0.63786 (6)	0.93385 (12)	0.00762 (13)	

### Atomic displacement parameters (Å^2^)

**Table:**

	*U*^11^	*U*^22^	*U*^33^	*U*^12^	*U*^13^	*U*^23^
Ni1	0.00486 (9)	0.00326 (8)	0.00613 (9)	0.000	−0.00056 (5)	0.000
Fe1	0.00264 (10)	0.00397 (11)	0.00455 (11)	0.000	0.000	−0.00016 (8)
Ca1	0.01508 (18)	0.01319 (18)	0.00735 (16)	0.000	0.000	0.000
P1	0.00450 (14)	0.00307 (14)	0.00398 (14)	0.000	−0.00041 (9)	0.000
P2	0.00320 (17)	0.00246 (17)	0.00414 (18)	0.000	0.000	0.000
O1	0.0045 (3)	0.0054 (3)	0.0079 (3)	0.0006 (2)	−0.0021 (2)	−0.0004 (2)
O4	0.0049 (4)	0.0057 (4)	0.0070 (4)	0.000	−0.0023 (3)	0.000
O3	0.0082 (4)	0.0045 (4)	0.0075 (4)	0.000	0.000	−0.0024 (3)
O2	0.0102 (3)	0.0069 (3)	0.0057 (3)	0.0018 (2)	0.0001 (2)	−0.0020 (2)

### Geometric parameters (Å, º)

**Table:**

Ni1—O1	2.0498 (8)	Ca1—O2^xi^	2.5987 (8)
Ni1—O1^i^	2.0499 (8)	Ca1—O2^xii^	2.5987 (8)
Ni1—O4	2.0529 (8)	Ca1—O2^xiii^	2.5987 (8)
Ni1—O4^ii^	2.0529 (8)	Ca1—O4^xiv^	2.5990 (12)
Ni1—O2^iii^	2.0841 (8)	Ca1—O4^xv^	2.5990 (12)
Ni1—O2^iv^	2.0841 (8)	Ca1—P2	3.1758 (7)
Fe1—O1^ii^	1.9504 (7)	Ca1—P2^xv^	3.2647 (7)
Fe1—O1^v^	1.9504 (7)	P1—O2^i^	1.5233 (8)
Fe1—O1^vi^	1.9504 (7)	P1—O2	1.5233 (8)
Fe1—O1^vii^	1.9504 (7)	P1—O1^i^	1.5719 (8)
Fe1—O3^viii^	2.0822 (11)	P1—O1	1.5719 (8)
Fe1—O3	2.0822 (11)	P2—O3	1.5259 (11)
Ca1—O3	2.5832 (12)	P2—O3^ix^	1.5259 (11)
Ca1—O3^ix^	2.5832 (12)	P2—O4^ix^	1.5498 (11)
Ca1—O2^x^	2.5987 (8)	P2—O4	1.5498 (11)
			
O1—Ni1—O1^i^	70.58 (4)	O2^x^—Ca1—O2^xi^	173.27 (4)
O1—Ni1—O4	171.24 (3)	O3—Ca1—O2^xii^	77.42 (2)
O1^i^—Ni1—O4	103.13 (3)	O3^ix^—Ca1—O2^xii^	108.72 (2)
O1—Ni1—O4^ii^	103.13 (3)	O2^x^—Ca1—O2^xii^	110.65 (3)
O1^i^—Ni1—O4^ii^	171.24 (3)	O2^xi^—Ca1—O2^xii^	68.92 (3)
O4—Ni1—O4^ii^	83.76 (5)	O3—Ca1—O2^xiii^	108.72 (2)
O1—Ni1—O2^iii^	90.33 (3)	O3^ix^—Ca1—O2^xiii^	77.42 (2)
O1^i^—Ni1—O2^iii^	92.27 (3)	O2^x^—Ca1—O2^xiii^	68.92 (3)
O4—Ni1—O2^iii^	83.75 (4)	O2^xi^—Ca1—O2^xiii^	110.65 (3)
O4^ii^—Ni1—O2^iii^	93.87 (4)	O2^xii^—Ca1—O2^xiii^	173.27 (4)
O1—Ni1—O2^iv^	92.27 (3)	O3—Ca1—O4^xiv^	141.08 (2)
O1^i^—Ni1—O2^iv^	90.33 (3)	O3^ix^—Ca1—O4^xiv^	141.08 (2)
O4—Ni1—O2^iv^	93.87 (4)	O2^x^—Ca1—O4^xiv^	64.19 (3)
O4^ii^—Ni1—O2^iv^	83.75 (4)	O2^xi^—Ca1—O4^xiv^	109.37 (3)
O2^iii^—Ni1—O2^iv^	176.81 (4)	O2^xii^—Ca1—O4^xiv^	109.37 (3)
O1^ii^—Fe1—O1^v^	180.0	O2^xiii^—Ca1—O4^xiv^	64.19 (3)
O1^ii^—Fe1—O1^vi^	85.81 (5)	O3—Ca1—O4^xv^	141.08 (2)
O1^v^—Fe1—O1^vi^	94.19 (5)	O3^ix^—Ca1—O4^xv^	141.08 (2)
O1^ii^—Fe1—O1^vii^	94.19 (5)	O2^x^—Ca1—O4^xv^	109.37 (3)
O1^v^—Fe1—O1^vii^	85.81 (5)	O2^xi^—Ca1—O4^xv^	64.19 (3)
O1^vi^—Fe1—O1^vii^	180.0	O2^xii^—Ca1—O4^xv^	64.19 (3)
O1^ii^—Fe1—O3^viii^	85.29 (3)	O2^xiii^—Ca1—O4^xv^	109.37 (3)
O1^v^—Fe1—O3^viii^	94.71 (3)	O4^xiv^—Ca1—O4^xv^	55.60 (5)
O1^vi^—Fe1—O3^viii^	94.71 (3)	O2^i^—P1—O2	112.08 (6)
O1^vii^—Fe1—O3^viii^	85.29 (3)	O2^i^—P1—O1^i^	116.00 (4)
O1^ii^—Fe1—O3	94.71 (3)	O2—P1—O1^i^	107.24 (4)
O1^v^—Fe1—O3	85.29 (3)	O2^i^—P1—O1	107.24 (4)
O1^vi^—Fe1—O3	85.29 (3)	O2—P1—O1	116.00 (4)
O1^vii^—Fe1—O3	94.71 (3)	O1^i^—P1—O1	97.76 (6)
O3^viii^—Fe1—O3	180.000 (10)	O3—P2—O3^ix^	107.35 (9)
O3—Ca1—O3^ix^	56.84 (5)	O3—P2—O4^ix^	111.66 (3)
O3—Ca1—O2^x^	77.42 (2)	O3^ix^—P2—O4^ix^	111.66 (3)
O3^ix^—Ca1—O2^x^	108.72 (2)	O3—P2—O4	111.66 (3)
O3—Ca1—O2^xi^	108.72 (2)	O3^ix^—P2—O4	111.66 (3)
O3^ix^—Ca1—O2^xi^	77.42 (2)	O4^ix^—P2—O4	102.91 (9)

Symmetry codes: (i) −*x*+3/2, *y*, −*z*+3/2; (ii) −*x*+3/2, −*y*+1/2, −*z*+3/2; (iii) *x*, −*y*+1, −*z*+2; (iv) −*x*+3/2, −*y*+1, *z*−1/2; (v) *x*−1/2, *y*−1/2, *z*−1/2; (vi) −*x*+3/2, *y*−1/2, *z*−1/2; (vii) *x*−1/2, −*y*+1/2, −*z*+3/2; (viii) −*x*+1, −*y*, −*z*+1; (ix) −*x*+1, −*y*+1/2, *z*; (x) −*x*+1, *y*−1/2, −*z*+1; (xi) *x*, −*y*+1, −*z*+1; (xii) *x*, *y*−1/2, −*z*+1; (xiii) −*x*+1, −*y*+1, −*z*+1; (xiv) −*x*+1, −*y*+1/2, *z*−1; (xv) *x*, *y*, *z*−1.
